# Inhibin B and AMH for Diagnosis of Hypogonadotropic Hypogonadism in Boys Under 1 Year of Age: A Case-control Study

**DOI:** 10.1210/clinem/dgaf219

**Published:** 2025-04-07

**Authors:** Tifenn Gueguen, Laetitia Martinerie, Sarah Castets, Vanessa Menut, Carine Villanueva, Anne Sophie Lambert, Kevin Perge, Natacha Bouhours-Nouet, Lucie Levaillant, Tristan Avril, Dominique Simon, Marc de Kerdanet, Najiba Lahlou, Sabine Baron, Rachel Reynaud, Marc Nicolino, Claire Bouvattier, Regis Coutant

**Affiliations:** Department of Pediatric Endocrinology, University Hospital, Angers 49100, France; Reference Center for Rare Pituitary Diseases, CHU Angers, Angers 49100, France; Pediatric Endocrinology Department, CHU Robert Debré, Assistance Publique-Hôpitaux de Paris, Paris 75019, France; Reference Center for Rare Disease CRMR de la Croissance et du Développement, CHU Robert Debré, Paris 75019, France; Université Paris Cité, Paris 75013, France; Department of Pediatric Endocrinology, Assistance Publique-Hôpitaux de Marseille, Marseille 13005, France; Reference Center for Rare Pituitary Diseases, CHU Marseille, Marseille 13005, France; Department of Pediatric Endocrinology, University Hospital, Nantes 44000, France; Department of Pediatric Endocrinology, Civil Hospices of Lyon, Lyon 69500, France; Reference Center for Rare Pituitary Diseases, CHU Lyon, Lyon 69500, France; Pediatric Endocrinology Department, CHU Bicetre, Assistance Publique-Hôpitaux de Paris, Le Kremlin Bicetre 94270, France; Paris Saclay University, Gif-sur-Yvette 91190, France; Reference Center for Rare Disease CRMR Dev Gen, Le Kremlin Bicêtre, Paris 94270, France; Department of Pediatric Endocrinology, Civil Hospices of Lyon, Lyon 69500, France; Reference Center for Rare Pituitary Diseases, CHU Lyon, Lyon 69500, France; Department of Pediatric Endocrinology, University Hospital, Angers 49100, France; Reference Center for Rare Pituitary Diseases, CHU Angers, Angers 49100, France; Department of Pediatric Endocrinology, University Hospital, Angers 49100, France; Reference Center for Rare Pituitary Diseases, CHU Angers, Angers 49100, France; Pediatric Endocrinology Department, CHU Robert Debré, Assistance Publique-Hôpitaux de Paris, Paris 75019, France; Reference Center for Rare Disease CRMR de la Croissance et du Développement, CHU Robert Debré, Paris 75019, France; Université Paris Cité, Paris 75013, France; Pediatric Endocrinology Department, CHU Robert Debré, Assistance Publique-Hôpitaux de Paris, Paris 75019, France; Reference Center for Rare Disease CRMR de la Croissance et du Développement, CHU Robert Debré, Paris 75019, France; Université Paris Cité, Paris 75013, France; Department of Pediatric Endocrinology, University Hospital, Rennes 35000, France; Department of Hormonology, BPR Specialized Analyses, Pannes 45700, France; Department of Pediatric Endocrinology, University Hospital, Nantes 44000, France; Department of Pediatric Endocrinology, Assistance Publique-Hôpitaux de Marseille, Marseille 13005, France; Reference Center for Rare Pituitary Diseases, CHU Marseille, Marseille 13005, France; Department of Pediatric Endocrinology, Civil Hospices of Lyon, Lyon 69500, France; Reference Center for Rare Pituitary Diseases, CHU Lyon, Lyon 69500, France; Pediatric Endocrinology Department, CHU Bicetre, Assistance Publique-Hôpitaux de Paris, Le Kremlin Bicetre 94270, France; Paris Saclay University, Gif-sur-Yvette 91190, France; Reference Center for Rare Disease CRMR Dev Gen, Le Kremlin Bicêtre, Paris 94270, France; Department of Pediatric Endocrinology, University Hospital, Angers 49100, France; Reference Center for Rare Pituitary Diseases, CHU Angers, Angers 49100, France

**Keywords:** male, AMH, testosterone, inhibin B, hypogonadotropic hypogonadism, infant

## Abstract

**Context:**

Congenital hypogonadotropic hypogonadism (CHH) in infant boys is a rare disorder that can manifest as micropenis and/or cryptorchidism. Mini-puberty is considered a window of opportunity for CHH diagnosis and treatment. The lack of testosterone (T) elevation during this period is the gold standard for CHH diagnosis, but hormonal evaluation is not always available at this time.

**Objectives:**

The aim was to compare inhibin B (INHB), anti-Müllerian hormone (AMH), T, LH, and FSH between infant boys (1 to 365 days) with micropenis and/or cryptorchidism due to isolated CHH (iCHH), CHH as part of combined pituitary hormone deficiency (CPHD), or of idiopathic origin (controls) and to determine discriminating cutoffs for CHH diagnosis based on sensitivity (Se) and specificity (Sp).

**Methods:**

This multicenter study from 7 University Hospitals in France included 138 boys aged 0 to 12 months (58 with iCHH, including 28 with a positive molecular diagnosis, 32 with CPHD, and 48 controls). Four periods of interest were studied: between 1 to 4 days, 15 to 65 days (early mini-puberty, corresponding to the T peak), 66 to 179 days (late mini-puberty), and 180 to 365 days (post mini-puberty).

**Results:**

Out of mini-puberty, the best-discriminating hormones were INHB between 1 to 4 days (Se/Sp 100%/75% at 150 pg/mL and 89%/100% at 85 pg/mL) and INHB and AMH after 180 days (INHB: Se/Sp 100%/100% at 100 pg/mL; AMH: Se/Sp 100%/92% at 600 pmol/L, and 75%/100% at 370 pmol/L). INHB and/or AMH discriminating performances were good (area under the receiver operating characteristic curve ≥ 0.95) across all 4 periods.

**Conclusion:**

Inhibin B and/or AMH can be used to diagnose CHH in boys < 1 year of age.

There are 3 physiological waves of hypothalamic-pituitary-gonadal (HPG) axis activity over the lifetime. The first occurs during fetal life, the second (“mini-puberty”) in the first months after birth, and the third at puberty (1, 2). The fetal HPG axis is functional from 12 weeks of gestation and contributes to gonadal androgen production in boys with placental human chorionic gonadotropin (hCG) (3). This allows the growth of the penis and the inguinoscrotal testicular descent during the second and third trimester of pregnancy. Therefore, the integrity of the HPG axis is crucial for the normal development of male external genitalia.

High placental estrogen levels at the end of pregnancy suppress the fetal HPG axis (3). During the first hours after birth, there is an early and fleeting peak of LH and testosterone (T), possibly linked to the interruption of this negative feedback control (3, 4). The second major phase of gonadotropic activation occurs in mini-puberty between 1 and 3 months of age (1-3). This activation stimulates the proliferation of Sertoli and germ cells and may be important for future fertility. Leydig cell production of T peaks at 1 month and then declines to half-peak levels around 4 months of life (5). Sertoli cells represent the main testicular contingent in childhood and support a limited number of germ cells (3). Sertoli cell production of anti-Müllerian hormone (AMH) and inhibin B (INHB) peaks at 4 to 5 months, then INHB concentrations decline to half-peak levels around 1 year (5, 6). At the same time, AMH concentrations decline very slowly during infancy. Mini-puberty is associated with an increase in testis volume (7).

Congenital hypogonadotropic hypogonadism (CHH) results from a decrease in hypothalamic GnRH and/or pituitary gonadotropin production (8). It occurs in between 1/4000 and 1/30 000 male births (3, 8). The diagnosis of CHH is usually and easily made at the end of adolescence-early adulthood, where the HPG axis is generally activated in all subjects because it results in absent or incomplete pubertal virilization and testis growth and low concentrations of gonadotropins and T compared to adult normal values (8). At birth and in the first months, CHH can result in micropenis (20% of CHH) and cryptorchidism (50% of CHH) (3). However, these anomalies are found in 5% of male newborns, and the diagnosis of CHH may represent a challenge at this time (3, 9). It is accessible during mini-puberty because of low levels of FSH, LH, and T compared to established standards reflecting the physiological transient activation of the HPG axis. However, case series describing the hormonal phenotype of CHH boys at this time are few, corresponding to less than 50 male subjects (10-19). Furthermore, in clinical practice, it is expected to see boys with suspected CHH out of mini-puberty: they may be referred in the first few days of life due to a neonatal diagnosis of abnormal genitalia or after 2 months if questions about the appearance of the genitalia arose lately [ie, when the gonadotropin and T peak of mini-puberty has already occurred in most boys (5)]. While many cases of young boys diagnosed with CHH have been reported, there has not been, to our knowledge, a systematic evaluation of pituitary gonadotropins (FSH and LH) or testicular hormones (T, AMH, INHB) measurements for diagnosing CHH during this period.

The main aim of this multicenter study, carried out in 7 French university hospitals, was to compare the hormonal profiles (LH, FSH, T, AMH, INHB) of 138 boys aged 0 to 12 months with cryptorchidism and/or micropenis, according to final diagnosis [isolated CHH (iCHH) or CHH as part of combined pituitary hormone deficiency (CPHD), idiopathic minor genital anomaly] and age (1-4 days, ie, during maternity stay, during early mini-puberty between 15 days and 2 months, during late mini-puberty between 2 and 6 months, and between 6 months and 1 year). For establishing a conclusive diagnosis of CHH, the boys were either studied during mini-puberty and/or had a positive molecular diagnosis for CHH and/or had anosmia and absent puberty at adolescence.

## Methods

### Patients

We studied 138 boys aged less than 1 year who presented with cryptorchidism and/or micropenis (20) and were followed in the Pediatric Endocrinology Department from 1 of 7 French university hospitals (Angers, Lyon, Marseille, Nantes, Paris Robert Debré, Paris Bicêtre, Rennes) between 2000 and 2022. This is a retrospective study based on clinical and biological data from these infant boys who were suspected of CHH in the first year of life.

**Figure 1.**
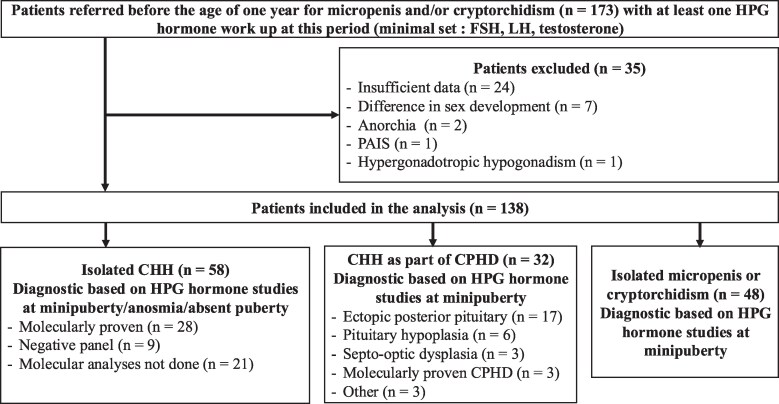
Flowchart of the study. Abbreviations: CPHD, combined pituitary hormone deficiency; HPG, hypothalamic-pituitary-gonad; PAIS, partial androgen insensitivity syndrome.

Inclusion criteria were to be male, aged less than 1 year, with micropenis and/or cryptorchidism, and with several hormonal measurements with a minimal set comprising T, LH, and FSH. Exclusion criteria were primary testicular insufficiency (FSH levels > 4.5 IU/L during mini-puberty, corresponding to + 2 SD score) (5, 6), differences in sexual development, and multiple malformations suggesting severe syndromic involvement. Subjects with CHH as part of CPHD were included. The flow chart is presented in Fig. 1.

The collection of personal data from the medical records for research purposes requested only the nonopposition from the children and their families, in agreement with French regulations (loi Jardé). Angers University Hospital's ethics committee approved the study (2023-031). The anonymous database was declared to the French Data Protection Authority (reference ar23-0052v0).

### Study Procedure

Clinical data were collected: gestational age, birth weight, length, head circumference, weight and length at the first medical consultation, penile length and width at the first medical consultation, presence of cryptorchidism, and testicular length and width. Testicular volume was estimated by the formula width × length^2^ × π/6 (5). Other malformations were recorded.

Circulating FSH, LH, T, AMH, and INHB levels were recorded. Other pituitary axes were investigated: cortisol, ACTH, dehydroepiandrosterone sulfate, TSH, free T4, free T3, IGF-1, and GH levels. The circulating T in response to an hCG test was recorded when performed (21). Different protocols were used for the hCG test: 6 × 1500 IU hCG IM or 2 × 125 µg recombinant human hCG subcutaneously, with T measurement 24 hours after the last injection (21).

#### Positive diagnosis of iCHH

Fifty-eight patients were diagnosed with iCHH based on 4 criteria (Supplementary Table 1) (22): (1) T levels were < 2.4 nmol/L (−2 SD score) during early mini-puberty (and FSH levels were < 4.5 IU/L, + 2 SD score at mini-puberty, ruling out primary testicular insufficiency), in agreement with normative values from Busch et al (5, 6) (n = 44) and/or (2) a molecularly proven cause of iCHH was found (n = 28) (see later discussion) (3, 8); (3) abnormal olfaction was diagnosed later in life (hyposmia/anosmia and abnormal olfactory bulbs/tracts on cranial magnetic resonance imaging (n = 11) (23); or (4) puberty was absent at adolescence (n = 3). Most of the boys were positive for at least 2 of these criteria. For some, this was, in addition, confirmed because the T response to hCG was < 1 ng/mL after mini-puberty (2-12 months) (21). A specific molecular diagnosis of iCHH was positive for 28 of 37 boys (76%), the gene panel for iCHH was negative for 9 out of 37 (see details of the individual diagnostic criteria in Supplementary Table 1 and of the gene panel in Supplementary Table 2) (22), and 21 of the 58 iCHH boys did not undergo molecular diagnosis with a gene panel (3, 8, 24).

#### Positive diagnosis of CHH as part of CPHD

Thirty-two patients were classified with CHH as part of CPHD when they had the same clinical and hormonal features as the iCHH group, combined with a deficiency of at least 1 other pituitary hormone. Seventeen patients (53% of this group) had ectopic posterior pituitary gland at cranial magnetic resonance imaging (MRI), and 3 (9% of this group) had septo-optic dysplasia. Six had severe pituitary hypoplasia at cranial MRI performed before the age of 1 month [sagittal pituitary height < 1 mm and absence of T1-weighted pituitary hypersignal (25)]. Three had an identified molecular cause of CPHD (see Results section).

#### Isolated micropenis and/or cryptorchidism = controls

Forty-eight boys were diagnosed with isolated micropenis and/or cryptorchidism when T levels during early mini-puberty were > 2.4 mmol/L (ie, −2 SD score) (5, 6) (and FSH was < 4.5 IU/L), and no pituitary hormone deficiency was found. For 3 boys, the diagnosis was ascertained because they developed spontaneous puberty in adolescence.

### Analytical Determinations

INHB was measured with 1 of 2 assays, with similar performances: (1) a solid-phase sandwich assay using Oxford Bioinnovation reagents (Diagnostic Systems Laboratories, distributed by Beckman-Coulter, Villepinte, France) (Oxford Bio-Innovation Cat# MCA1312KZZ, RRID:AB_2800544); the intra- and interassay coefficients of variation (CV) were 5.7% and 12%, respectively, at 112 pg/ml, and the sensitivity was 6 pg/ml; (2) a solid-phase sandwich assay using Ansh Labs reagents (Webster, TX) (Ansh Labs Cat# AL-107, RRID:AB_2783661); the intra- and interassay CV were 3% and 4.6%, at 110 pg/mL, and the sensitivity was 7 pg/mL.

AMH was measured with 1 of 2 assays, which performed similarly: (1) a solid-phase sandwich assay using Oxford Bioinnovation reagents (Diagnostic Systems Laboratories, distributed by Beckman-Coulter) (Beckman Coulter Cat# A79765, RRID:AB_2800500); the intra- and interassay CVs were 1.4% and 2.5%, respectively, at 557 pmol/L, and the sensitivity was 0.7 pmol/L; (2) a solid-phase sandwich assay using Ansh Labs reagents (Ansh Labs Cat# AL105, RRID:AB_2783659); intra- and interassay CVs were 2.5% and 5.1%, respectively, at 650 pmol/L, and the sensitivity was 1.3 pmol/L.

FSH and LH were measured with 3 assays: (1) a time-resolved fluorometric assay using Delfia reagents (Perkin Elmer Life Sciences, Courtaboeuf, France) (FSH Kit AutoDelfia, PerkinElmer Cat# B017-201, RRID:AB_2783738) (LH spec Kit AutoDelfia, PerkinElmer Cat# B031-101, RRID:AB_2783737); in the FSH assay, the intra- and interassay CV were 1.2% and 3.9%, respectively; the intra- and interassay CVs in the LH assay were 1.4% and 2.6%, respectively; and the sensitivity was 0.01 IU/L for both assays; (2) a sensitive chemiluminescent immunometric assay (Centaur, Siemens) (LH, Siemens Cat# 01756298, RRID:AB_2895592) (FSH, Siemens Cat# 04912924, RRID:AB_2895593); the intra- and inter-assays CV were, respectively, 2.9% and 2.7% for FSH and 2.3% and 1.5% for LH, and the sensitivities were 0.3 IU/L for FSH and 0.07 IU/L for LH; (3) a chemiluminescent immunometric assay (DXi600 Beckman Coulter) (LH, Beckman Coulter Cat# 33510, RRID:AB_2750984) (FSH, Beckman Coulter Cat# 33520, RRID:AB_2750983); the intra- and inter-assays CV were, respectively, 4.6 and 5.2% for FSH and 3.5 and 6.4% for LH, and the detection limits were 0.2 IU/L for FSH and 0.2 IU/L for LH.

Total plasma T concentration was measured with 1 of 2 methods: (1) liquid chromatography-mass spectrometry (Quatro Premier, Waters, Guyancourt, France); the intra- and interassays CV were 5.5 and 6.7%, respectively, and the sensitivity was 0.02 nmol/L; (2) a radioimmunoassay, after a steroid extraction step, using Orion reagents (Cis Bio International, Gif sur Yvette, France) (Abcam Cat# ab33863, RRID:AB_778304); the intra- and interassay CVs were 5.9% and 8.2%, and the sensitivity was 0.046 nmol/L. Accuracy was not different from that of the tandem mass spectrometric assay developed in the same laboratory, with the regression curve being T value measured by radioimmunoassay = 1.0 × T value measured by mass spectrometry + 0.0049.

Other hormones were measured using commercially available kits.

## Statistics

### Baseline Statistics

Continuous variables were expressed as medians (5th and 95th percentiles), and discrete variables were expressed as percentages. The precision for age was at the day level. Kruskal–Wallis, Mann–Whitney, chi-squared, and Fisher exact tests were used for comparisons.

### Receiver Operating Characteristic Curves

Receiver operating characteristic (ROC) curves were constructed to determine optimal baseline INHB, AMH, T, FSH, and LH cutoffs to distinguish CHH from isolated micropenis and/or cryptorchidism. Each test's area under the ROC curve was expressed as means and 95% confidence interval (CI). Four periods were studied: 1 to 4 days, between 15 and 65 days (early mini-puberty, corresponding to the 80% CI of the T peak) (5, 6), between 65 and 179 days (late mini-puberty, after the T peak), and between 180 days and 1 year (after mini-puberty). Based on INHB, AMH, T, FSH, and LH cutoffs, we calculated sensitivity and specificity (and 95% CIs) and likelihood ratios for the diagnosis of CHH [except for T during early mini-puberty, as it was a diagnostic criterion for CHH, so the T ROC area under the curve (AUC) was 1]. Sensitivity was defined as the proportion of patients with CHH having a hormone result below the cutoff. Specificity was defined as the proportion of patients with isolated micropenis and/or cryptorchidism having a hormone result above the cutoff. We emphasized hormonal cutoffs with the best sensitivity (minimal percentage of false negatives) and specificity (minimal percentage of false positives). We considered that the best values for sensitivity and specificity could not be associated with specificity and specificity below 50%, respectively (as a result, the best values did not always reach 100%). The combined pituitary hormone deficiency and hypogonadotropic hypogonadism groups had similar hormone levels (for the HPG axis), so they were combined into a single CHH group (see details in the Results section).

### Multivariate Approach

Univariate logistic regression analyses were used to determine the predictors of CHH. Then multiple backward logistic regression analyses were used to determine the best set of predictors. The Hosmer–Lemeshow statistics assessed the regression model's goodness of fit.

*P-*values <.05 were considered to be statistically significant. We used SPSS Statistics v25 (IBM Corp., Armonk, NY), Jamovi software version 2.3.18.0 (The Jamovi Project, 2022; https://www.jamovi.org), and GraphPad Prism 9 (GraphPad Software Inc.).

## Results

### Clinical and Hormonal Characteristics of the Patients

**Figure 2.**
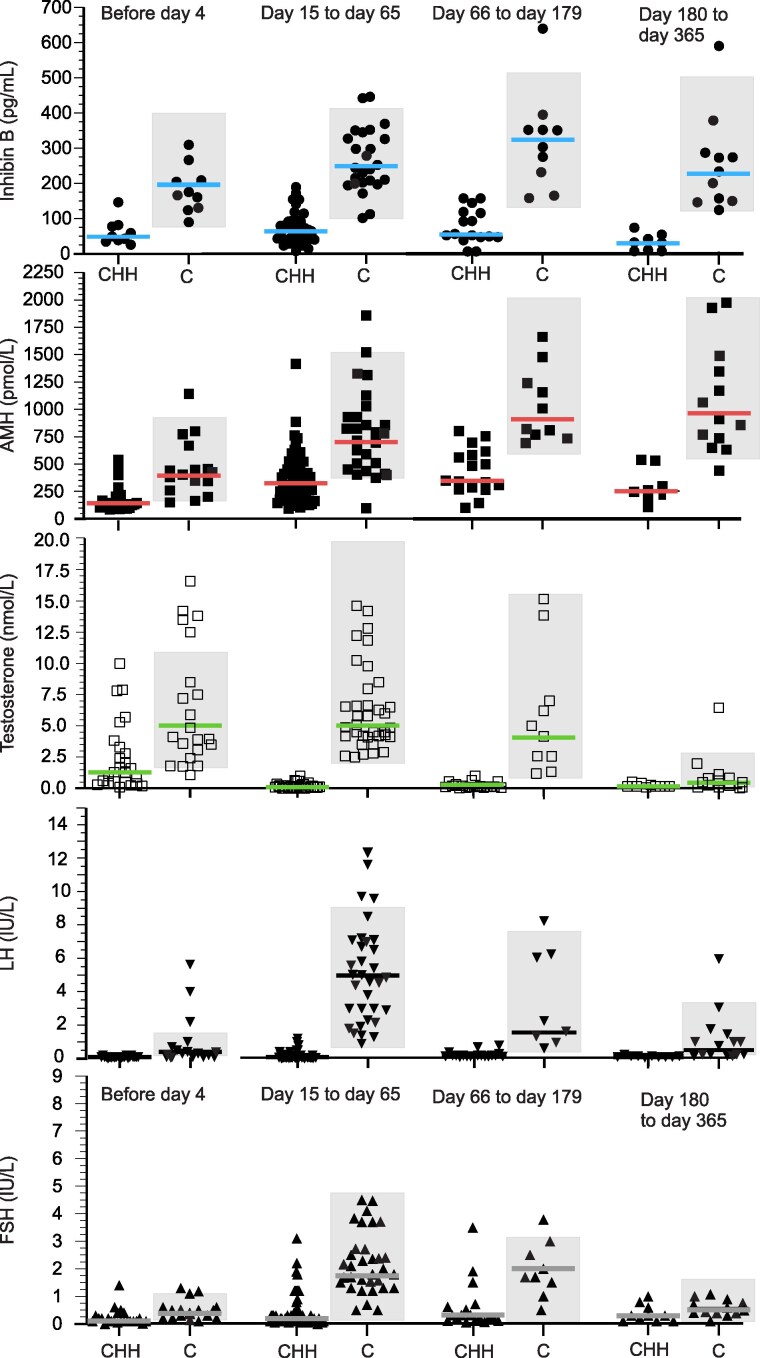
Inhibin B, AMH, testosterone, LH, and FSH in boys with CHH and isolated cryptorchidism/micropenis (C), 1 to 4 days, between 15 and 65 days, between 66 days and 179 days, and between 180 and 365 days. Black horizontal lines depict medians. The light gray rectangles represent the normal range of hormone concentration (3rd; 97th percentile) at the corresponding meantime, according to the literature (5, 6, 25, 26). Abbreviations: AMH, anti-Müllerian hormone; C, controls; CHH, congenital hypogonadotropic hypogonadism.

A total of 58 boys with iCHH, 32 boys with CHH as part of CPHD, and 48 control boys with isolated cryptorchidism and/or micropenis were included in the analysis. The clinical and hormonal characteristics are presented in Tables 1 and 2, as well as in Fig. 2.

**Table 1.** Clinical characteristics of the subjects

	iCHH (n = 58)	CHH, as part of CPHD (n = 32)	Controls (n = 48)	*P*
Gestational age (weeks)	39 (35.8; 41.1)	40.5 (32.6; 42.0)*^a^*	39.0 (33.0; 41.0)	NS
Birth weight (kg)	3.24 (2.28; 3.79)	3.32 (1.60; 4.38)	3.14 (1.45; 4.21)	NS
Birth length (cm)	50.0 (43.9; 52.0)	50.0 (42.0; 52.0)	49.5 (44.3; 52.9)	NS
Birth HC (cm)	34.0 (31.1; 36.0)*^b^*	35.0 (29.7; 38.0)*^a^*	34.0 (31.1; 36.2)	.04
Age at referral (days)	30.0 (1.4; 251)*^a,b^*	2.5 (1.3; 65)	5.0 (1.2; 65)	<.001
Micropenis n (%)	58 (100)	32 (100)	26 (54)	
Penis length (cm)	1.7 (1.0; 2.4)*^a^*	1.5 (1.0; 2.4)*^a^*	2.7 (1.3; 3.4)	< .001
Penis width (cm)	0.8 (0.5; 1.1)*^a^*	0.7 (0.5; 1.0)*^a^*	1.0 (0.6; 1.4)	.02
Cryptorchidism n (%)	33 (57)	14 (44)	26 (54)	
Unilateral	9 (16)	5 (16)	2 (4)	
Bilateral	24 (41)	9 (28)	24 (50)	
Testis volume (ml)	0.26 (0.10; 1.69)	0.26 (0.10; 1.07)	0.38 (0.10; 1.50)	NS

Controls were isolated cryptorchidism and/or micropenis. Measurements in the neonatal period were not performed in cord blood. Median (5th; 95th percentile).

Abbreviations: AMH, anti-Müllerian hormone; CHH, congenital hypogonadotropic hypogonadism; CPHD, combined pituitary hormone deficiency; HC, head circumference; iCHH, isolated congenital hypogonadotropic hypogonadism; NS, nonsignificant.

^*a*^< .05 for the Mann–Whitney test (compared with controls).

^*b*^ *P* < .05 for the Mann–Whitney test (compared with CHH as part of CPHD).

**Table 2.** Hormonal characteristics of the subjects

	iCHH (n = 58)	CHH, as part of CPHD (n = 32)	Controls (n = 48)	*P*
1-4 days Day	n = 15 2.0 (1.0; 4.0)	n = 21 2.0 (1.0; 4.0)	n = 22 2.0 (1.0; 4.0)	NS
FSH (IU/L)	0.10 (0.07; 0.55)*^a^*	0.15 (0.01; 0.85)*^a^*	0.40 (0.05; 1.30)	.02
LH (IU/L)	0.10 (0.05; 0.15)*^a^*	0.10 (0.01; 0.20)*^a^*	0.35 (0.05; 5.63)	< .001
Testosterone (nmol/L)	2.1 (0.2; 8.7)*^a^*	0.7 (0.2; 4.7)*^a^*	4.9 (0.6; 16.6)	.02
AMH (pmol/L)	97 (64; 315)*^b^*	120 (62; 355)*^b^*	385 (150; 1120)	< .001
Inhibin B (ng/mL)	51 (40; 75)*^b^*	49 (28; 134)*^b^*	190 (90; 310)	.007
Mini-puberty (15-65 days) Day	n = 44 35 (18; 60)	n = 32 45 (15; 60)	n = 48 35 (16; 60)	NS
FSH (IU/L)	0.30 (0.10; 1.9)*^a,b^*	0.30 (0.05; 0.82)*^a^*	1.80 (0.50;4.48)	< .001
LH (IU/L)	0.07 (0.03; 0.78)*^a^*	0.10 (0.00; 0.72)*^a^*	4.80 (1.07; 11.93)	< .001
Testosterone (nmol/L)	0.4 (0.0; 1.4)*^a,b^*	0.1 (0.0; 1.0)*^a^*	5.0 (2.7; 14.3)	< .001
AMH (pmol/L)	270 (119; 527)*^a,b^*	367 (145; 903)*^a^*	700 (195; 1720)	< .001
Inhibin B (ng/mL)	63 (25; 117)*^a^*	64 (19; 317)*^a^*	252 (105; 444)	< .001
Between 66 and 179 days Day	n = 17 90 (75; 130)	n = 5 105 (90; 120)	n = 10 120 (75; 130)	NS
FSH (UI/L)	0.30 (0.05; 1.10)*^a^*	0.20 (0.04; 0.60)*^a^*	2.0 (0.40; 3.80)	< .001
LH (UI/L)	0.10 (0.04; 0.70)*^a^*	0.10 (0.01; 0.20)*^a^*	1.7 (0.20; 8.2)	< .001
Testosterone (nmol/L)	0.13 (0.00; 1.00)*^a^*	0.10 (0.06; 0.20)*^a^*	4.2 (0.8; 15.10)	< .001
AMH (pmol/L)	487 (146; 805)*^a^*	223 (103; 343)*^a^*	907 (600; 1660)	< .001
Inhibin B (ng/mL)	74 (38; 158)*^a,b^*	19 (6; 67)*^a^*	330 (140; 645)	< .001
Between 180 and 365 days Day	n = 10 240 (180; 360)	n = 5 240 (180; 300)	n = 14 239 (180; 330)	NS
FSH (UI/L)	0.30 (0.10; 1.00)*^a^*	0.30 (0.10; 0.60)*^a^*	0.55 (0.10; 1.10)	0.18
LH (UI/L)	0.05 (0.01; 0.20)*^a^*	0.10 (0.01; 0.20)*^a^*	0.45 (0.10; 5.8)	.004
Testosterone (nmol/L)	0.10 (0.00; 0.45)*^a^*	0.10 (0.01; 0.20)*^a^*	0.50 (0.00; 6.40)	.15
AMH (pmol/L)	231 (109; 534)*^a^*	420 (300; 541)*^a^*	1033 (443; 1984)	< .001
Inhibin B (ng/mL)	20 (6; 53)*^a^*	52 (31; 73)*^a^*	232 (122; 589)	< .001

Controls were isolated cryptorchidism and/or micropenis. Measurements in the neonatal period were not performed in cord blood. Median (5th; 95th percentile).

Abbreviations: AMH, anti-Müllerian hormone; CHH, congenital hypogonadotropic hypogonadism; CPHD, combined pituitary hormone deficiency; HC, head circumference; iCHH, isolated congenital hypogonadotropic hypogonadism; NS, nonsignificant.

^*a*^ < .05 for the Mann–Whitney test (compared with controls).

^*b*^ *P* < .05 for the Mann–Whitney test (compared with CHH as part of CPHD).

Of the 58 patients with iCHH, 36 had Kallmann syndrome. It was molecularly proven in 25 (8 CHD7 mutations, 6 FGFR1 mutations, 7 ANOS1 mutations, 2 PROKR2 mutations, 1 digenic SOX10/SEMA3F mutation, and 1 digenic ROBO3/IGFS10 mutation with anosmia and later absent puberty). The other 11 had anosmia and/or abnormal olfactory tracts/bulbs on cranial MRI. One patient had a GnRH receptor mutation, 1 had a KISS1R mutation, and 1 had a digenic TACR3/WDR11 mutation. In total, the iCHH gene panel was performed in 37 patients and was positive in 28 patients (76%), and the remaining 21 did not benefit from molecular diagnosis. The clinical and hormonal characteristics of the boys with molecularly proven iCHH are presented in Supplementary Table 3 (22).

Of the 32 patients with CHH as part of CPHD, 17 had an ectopic posterior pituitary with pituitary stalk interruption on cranial MRI, 3 had septo-optic dysplasia, 6 had severe pituitary hypoplasia (see Methods section), 3 had syndromic CPHD (1 boy with GLI3 mutation, 1 with GLI2 mutation, and 1 with SOX2 mutation), and 3 had CPHD with normal MRI and no identified molecular diagnosis.

Finally, in the control group, 22 patients had isolated micropenis, 22 had isolated cryptorchidism, and 4 had an association of micropenis and cryptorchidism.

The clinical and hormonal characteristics of the boys with iCHH and CHH as part of CPHD significantly differed from the controls at each of the 3 periods (Table 1) (Fig. 2). Conversely, there was no significant difference between boys with iCHH and CHH within CPHD (Table 1), who were grouped for further analyses (separate analyses are provided in Supplementary Table 4 and Table 5) (22).

### ROC Curves for Single Hormone Measurements

#### Between 1 and 4 days

Before the fourth day, the ROC curve AUC for differentiating CHH from controls was the greatest for INHB [AUC 0.97 (95% CI .90-1, *P* < .001)]. For INHB, the best sensitivity (100%) was obtained for a cutoff of 150 pg/mL (specificity 75%) and the best specificity (100%) for a cutoff of 85 pg/mL (sensitivity 89%). Hormones' ROC curve AUC, cutoffs, sensitivity, and specificity (and 95% CIs) and likelihood ratios are indicated in Table 3.

**Table 3.** Receiver operating characteristic curves for inhibin B, AMH, testosterone, FSH, and LH, differentiating CHH from isolated micropenis and/or cryptorchidism across 4 periods: 1-4 days, between 15 and 65 days (early mini-puberty), between 66 and 179 days (late mini-puberty), and between 180 days and 1 year (after mini-puberty). During early mini puberty, T was a diagnostic criterion for CHH, resulting in a T ROC curve AUC of 1. Hormonal cutoffs that exhibited the best sensitivity and specificity are highlighted

Period	ROC AUC (95% CI)	Cutoff	Sensitivity (%) (95% CI)	Specificity (%) (95% CI)	Likelihood ratio
**Day 1 to day 4**					
Inhibin B (pg/mL)	0.97 (.90-1.0)				
Best sensitivity		< 150	100 (70-100)	75 (41-96)	4.0
Best specificity		< 85	89 (57-99)	100 (68-100)	∞
AMH (pmol/L)	0.89 (.79-.99)				
Best sensitivity		< 390	91 (73-98)	53 (30-75)	2.0
Best specificity		<145	70 (49-84)	100 (80-100)	∞
Testosterone (nmol/L)	0.81 (.68-.94)				
Best sensitivity		< 3.9	80 (61-91)	58 (36-77)	1.9
Best specificity		<1.7	60 (41-77)	95 (76-100)	11.4
LH (IU/L)	0.86 (.73-.99)				
Best sensitivity		<0.25	100 (87-100)	63 (39-82)	2.7
Best specificity		<0.15	87 (68-95)	75 (51-90)	3.5
FSH (IU/L)	0.76 (.61-.92)				
Best sensitivity		< 0.40	78 (58-90)	56 (33-77)	1.8
Best specificity		<0.25	74 (53-87)	75 (51-90)	3.0
**Day 15-day 65**					
Inhibin B (pg/mL)	0.96 (.92-1.0)				
Best sensitivity		<190	98 (88-1.0)	85 (68-94)	6.6
Best specificity		< 100	82 (69-91)	100 (88-100)	∞
AMH (pmol/L)	0.84 (.74-.93)				
Best sensitivity		< 660	92 (82-96)	54 (35-71)	2.0
Best specificity		<395	64 (52-75)	92 (76-99)	8.4
Testosterone (nmol/L)	1.0				
Best sensitivity		< 2.4	100	100	
Best specificity					
LH (IU/L)	0.99 (.99-1.0)				
Best sensitivity		< 1.20	100 (95-100)	97 (83-100)	30.0
Best specificity		< 0.85	97 (90-99)	100 (89-100)	∞
FSH (IU/L)	0.93 (.87-.98)				
Best sensitivity		< 1.75	92 (83-97)	55 (38-71)	2.0
Best specificity		< 0.45	78 (67-87)	100 (89-100)	∞
**Day 66-day 179**					
Inhibin B (pg/mL)	0.97 (.92-1.0)				
Best sensitivity		< 180	100 (81-100)	80 (49-96)	5.0
Best specificity		< 130	81 (57-93)	100 (72-100)	∞
AMH (pmol/L)	0.93 (.85-1.0)				
Best sensitivity		< 806	100 (82-100)	60 (31-83)	2.5
Best specificity		<590	72 (49-88)	100 (72-100)	∞
Testosterone (nmol/L)	0.99 (.98-1.0)				
Best sensitivity		< 1.1	100 (84-100)	91 (62-100)	11.0
Best specificity		<0.72	95 (76-100)	100 (74-100)	∞
LH (IU/L)	0.98 (.92-1.0)				
Best sensitivity		<0.75	100 (84-100)	89 (56-99)	9
Best specificity		<0.15	75 (53-89)	100 (70-100)	∞
FSH (IU/L)	0.81 (.65-.97)				
Best sensitivity		< 2.0	85 (64-95)	56 (27-81)	1.91
Best specificity		<0.35	60 (39-78)	100 (70-100)	∞
**Day 180-day 365**					
Inhibin B (pg/mL)	1.0				
Best sensitivity		100	100 (68-	100 (74-	∞
Best specificity			100)	100)	
AMH (pmol/L)	0.98 (.93-1.0)				
Best sensitivity		< 600	100 (68-100)	92 (67-100)	13.0
Best specificity		<370	75 (41-96)	100 (77-100)	∞
Testosterone (nmol/L)	0.69 (.47-.91)	NS			
LH (IU/L)	0.92 (.81-1.0)				
Best sensitivity		<0.45	100 (72-100)	54 (29-77)	2.2
Best specificity		< 0.08	50 (24-76)	100 (77-100)	∞
FSH (IU/L)	0.75 (.54-.96)	NS			

When the ROC AUC was not significantly different from 0.5, cutoffs were not presented. We considered that the best values for sensitivity and specificity could not be associated with specificity and specificity below 50%, respectively (as a result, the best values did not always reach 100%).

Abbreviations: AMH, anti-Müllerian hormone; CHH, congenital hypogonadotropic hypogonadism; CI, confidence interval; NS, nonsignificant; ROC AUC, area under the receiver operating characteristic curve.

#### Between 15 and 65 days (early mini-puberty)

During early mini-puberty, the ROC curve AUC was the greatest for T (AUC 1, *P* < .001), as the consequence of the chosen methods for defining CHH, followed by LH [AUC 0.99 (95% CI .99-1, *P* < .001)] and INHB [AUC 0.96 (95% CI .92-1, *P* < .001)]. For LH, the best sensitivity (100%) was obtained for a cutoff of 1.2 IU/L (specificity 96%) and the best specificity (100%) for a cutoff of 0.85 IU/L (sensitivity 97%). For INHB, the best sensitivity (98%) was obtained for a cutoff of 190 pg/mL (specificity 85%) and the best specificity (100%) for a cutoff of 100 pg/mL (sensitivity 82%) (see Table 3 for detailed results).

#### Between 66 and 179 days (late mini-puberty)

Between 66 and 179 days, the ROC curve AUC was the greatest for T [AUC 0.99 (95% CI .98-1, *P* < .001)], followed by INHB [AUC 0.97 (95% CI .92-1, *P* < .001)] and AMH [AUC 0.93 (95% CI .85-1, *P* < .0001)]. For T, the cutoffs were lower than those determined in early mini-puberty: 1.1 nmol/L for 100% sensitivity (specificity 91%) and 0.72 nmol/L for 100% specificity (sensitivity 95%). For INHB, the best sensitivity (100%) was obtained for a cutoff of 180 pg/mL (specificity 80%) and the best specificity (100%) for a cutoff of 130 pg/mL (sensitivity 82%). For AMH, the best sensitivity (100%) was obtained for a cutoff of 806 pmol/L (specificity 60%) and the best specificity (100%) for a cutoff of 590 pmol/L (sensitivity 72%) (see Table 3 for detailed results).

#### Between 180 and 365 days (after mini-puberty)

Between 180 and 365 days, the ROC curve AUC was the greatest for INHB (AUC 1.0, *P* < .001), followed by AMH [AUC 0.98 (95% CI .93-1, *P* < .001)]. For INHB, the cutoff of 100 pg/mL was associated with 100% sensitivity and specificity. For AMH, the best sensitivity (100%) was obtained for a cutoff of 600 pmol/L (specificity 92%) and the best specificity (100%) for a cutoff of 370 pmol/L (sensitivity 75%). Other hormones' ROC curve AUCs were lower, likely because their peaks occur before 6 months in normal boys (Table 3).

#### Combined hormone measurements: multivariate model

We did not find any combination of HPG hormones significantly associated with a better prediction of CHH than the single hormone measurements (data not shown).

#### ROC curves for subjects with molecularly proven iCHH or with CHH as part of CPHD

Supplementary Tables 4 and 5 show the ROC curve analyses for subjects with molecularly proven iCHH and CHH as part of CPHD, respectively (22). The cutoffs for optimal sensitivity and specificity for these groups were close to those of the whole group of boys with CHH.

## Discussion

This study, based on a retrospective cohort of male infants with minor abnormalities of the external genitalia, has the potential to impact early diagnosis and treatment of CHH. We have highlighted significant differences in circulating hormone levels of the HPG axis between subjects with CHH and those with idiopathic micropenis or cryptorchidism, even out of the period of mini-puberty. This could lead to early diagnosis, interventions, and counseling that may improve outcomes for these infants.

In the present study, T levels were detectable from the very first days, significantly higher in controls compared to subjects with CHH, but it was not the best-discriminating hormone at this period. A threshold of 1.7 nmol/L provided a specificity of 95% (but a low sensitivity of 60%). The T range in controls before day 4 is similar to the T levels described in the literature in normal boys (26). The detectable T concentration likely resulted from the very transient activation of the HPG axis following the interruption of the negative feedback exerted by placental estrogens at the end of pregnancy (3, 4). Only 1 study reported T levels early after birth in 4 boys with CHH, with values less than 0.24 nmol/L (14). During early and late mini-puberty, however, T was the most discriminating hormone for the diagnosis of CHH. This could be awaited since T levels during early mini-puberty were the basis of CHH diagnosis in our study. Very different T levels were reported during early mini-puberty between normal boys, where the 95% CI ranged from 2.4 to 21 nmol/L (5, 6), and CHH boys, where T levels were mainly below 0.50 nmol/L (n = 23) (10-19). After 180 days, after the physiological T peak of mini-puberty, T no longer discriminates in diagnosing CHH since its level declines rapidly (5, 6). A diagnosis of CHH should, therefore, rely on other hormone measurements if a suspected boy is seen after this period.

INHB was the most discriminating hormone before day 4. A threshold of 150 pg/mL provided a sensitivity of 100% (for a fair specificity of 75%), while a threshold of 85 pg/mL provided a specificity of 100% (for a fair sensitivity of 89%). These thresholds can help to determine which male neonates need to be monitored during mini-puberty. Early postnatal INHB measurement likely reflects the first wave of Sertoli cell multiplication resulting from fetal HPG axis activation (3, 27-29). In the literature, INHB values were less than 100 pg/mL in CHH boys (n = 8) in line with our best sensitive cutoff (10, 14, 16). Between day 15 and day 65 and day 66 and day 179, an INHB cutoff of 100 and 130 pg/mL, respectively, provided 100% specificity (82% and 81% sensitivity). The 95% CI for INHB values in normal boys at the same period was 105 to 380 pg/mL (5, 6), which aligns with our best specific cutoff. In comparison, INHB values reported in a case series of CHH boys (totaling 46 cases) were less than 150 pg/mL (and less than 100 pg/mL in 85% of cases) (10-14, 16, 17, 19). Finally, INHB was the best-discriminating hormone between 180 and 365 days, with a threshold of 100 pg/mL providing a sensitivity of 100% (for a specificity of 100%). These results are consistent with the kinetics of INHB, whose peak response to the activation of mini-puberty occurs between 3 to 4 months, followed by a slow decline (5, 6). The 95% CI for INHB in normal boys reported in the literature at the same ages was 125 to 500 pg/mL (5, 6), which agrees with our specific cutoff. Similarly, INHB values reported in a few CHH boys between 6 months and 1 year (n = 6) were below 155 pg/mL (below 100 pg/mL in 67% of cases) (10, 11, 13, 19).

AMH showed the best discriminating power after day 179. AMH, like INHB, is produced by Sertoli cells (3). It increases in response to activation of the HPG axis, but its production is partly independent of FSH (3, 30-32) and is considered a reflection of Sertoli cell mass. It peaks at 4 months and then declines very slowly, with circulating AMH at 1 year of age still being 90% of the peak AMH value (5, 6). This kinetics is consistent with a strong discriminating power from 6 months, as 2 waves of Sertoli cells multiplication, 1 fetal and 1 at mini-puberty, should have occurred in normal boys but not in boys with CHH (33). Between 180 days and 1 year, a threshold of 600 pmol/L provided a sensitivity of 100% (for a specificity of 92%), while a threshold of 370 pmol/L provided a specificity of 100% (for a fair sensitivity of 75%). The 95% CI for AMH in normal boys at the same period ranged from 500 to 2400 pmol/L (5, 6, 34). Similarly, AMH values reported in a few CHH boys (n = 6) between 6 months and 1 year were below 600 pmol/L in 67% of cases (11, 13, 19).

Gonadotropin measurements were less discriminating in all periods, except for LH during mini-puberty. Out of the period of mini-puberty, physiological levels of gonadotropins are naturally low, close to the assays' lower detection limit. Ultrasensitive assays are needed to provide a discriminating power to gonadotropin measurements.

Our study has several limitations. The diagnosis of CHH was partly based on hormonal measurements during mini-puberty, which is a critical window for diagnosis (3, 35). However, this does not formally confirm the diagnosis, as only abnormal pubertal development could ultimately validate it. In addition, 47% of the iCHH boys also had a positive molecular diagnosis of CHH, and the hormone cutoffs were close when the ROC curve testing was restricted to this population. A second limitation was that the control population had idiopathic cryptorchidism and/or micropenis and, therefore, could not be considered normal, even if we carefully discarded boys with testicular insufficiency, differences in sex development, and polymalformative syndromes. Hormonal studies in cryptorchid boys of similar age have shown subtle changes in LH, FSH, and INHB values, though all within the normal range (36). In agreement, we also checked that the hormonal values found in this control group were within the range of those described in the normal boys population (5, 6, 26, 27, 29, 31, 34). The number of control boys referred to the tertiary centers participating in the current study was lower than expected (compared to the number of CHH boys), suggesting some bias in recruiting these normal subjects. It is likely that boys with a clearly normal hormonal profile were not referred, but considering these possibly overlooked boys would only enhance our sensitivity and specificity figures. Finally, this situation is precisely the one encountered in clinical practice, where it is helpful to discriminate between idiopathic abnormal genitalia (5% of male infants) and abnormal genitalia secondary to hormonal defects. Last, when we compared the thresholds provided by ROC curve testing in our study with the distribution and kinetics of the HPG hormones in normal male infants from the literature, we found that the cutoffs that provided the optimal specificity were close to the lower physiological distribution, as awaited from a group with a normal HPG axis (5, 6, 26, 27, 29, 31, 34). Finally, the main strength of the present multicenter study was the number of patients with CHH studied before the age of 1 year, the highest reported to our knowledge. This was also a limitation since hormone measurements were performed using several different assays due to the multicentric nature of our study. Although the assays used for AMH, INHB, and T measurements showed similar performances, this may have impacted the AUC ROC curves for discriminating CHH from controls.

In conclusion, since mini-puberty is associated with the second wave of multiplication of Sertoli and germ cells (1-3), it has been proposed to treat male boys with CHH in the first months of life with recombinant gonadotropins to mimic mini-puberty, with the hope of favoring later fertility (3, 10, 11). Our findings, namely the proposed hormone cutoffs for diagnosis of CHH before 1 year of age, if confirmed in a validation cohort, may help identify infants who could benefit from early treatment.

## Data Availability

Some or all datasets generated during and/or analyzed during the current study are not publicly available but are available from the corresponding author on reasonable request.
